# Supplementary material for: Exome sequencing and analysis of 44,028 British South Asians enriched for high autozygosity
Source: Nat Genet. 2026 Mar 27;58(4):821–30. doi: 10.1038/s41588-026-02553-7 (PMC13083238; doi:10.1038/s41588-026-02553-7)
Supplement: Supplementary file 2 — Reporting Summary [file 41588_2026_2553_MOESM2_ESM.pdf]

nature portfolio

Corresponding author(s): Hye In Kim
David A. van Heel
Hilary C. Martin

Last updated by author(s): Feb 1st, 2026

Reporting Summary

Nature Portfolio wishes to improve the reproducibility of the work that we publish. This form provides structure for consistency and transparency in reporting. For further information on Nature Portfolio policies, see our Editorial Policies and the Editorial Policy Checklist.

Statistics

For all statistical analyses, confirm that the following items are present in the figure legend, table legend, main text, or Methods section.

n/a	Confirmed	
☐	☒	The exact sample size (n) for each experimental group/condition, given as a discrete number and unit of measurement
☐	☒	A statement on whether measurements were taken from distinct samples or whether the same sample was measured repeatedly
☐	☒	The statistical test(s) used AND whether they are one- or two-sided<br>*Only common tests should be described solely by name; describe more complex techniques in the Methods section.*
☐	☒	A description of all covariates tested
☐	☒	A description of any assumptions or corrections, such as tests of normality and adjustment for multiple comparisons
☐	☒	A full description of the statistical parameters including central tendency (e.g. means) or other basic estimates (e.g. regression coefficient) AND variation (e.g. standard deviation) or associated estimates of uncertainty (e.g. confidence intervals)
☐	☒	For null hypothesis testing, the test statistic (e.g. F, t, r) with confidence intervals, effect sizes, degrees of freedom and P value noted<br>*Give P values as exact values whenever suitable.*
☒	☐	For Bayesian analysis, information on the choice of priors and Markov chain Monte Carlo settings
☒	☐	For hierarchical and complex designs, identification of the appropriate level for tests and full reporting of outcomes
☐	☒	Estimates of effect sizes (e.g. Cohen's d, Pearson's r), indicating how they were calculated

Our web collection on statistics for biologists contains articles on many of the points above.

Software and code

Policy information about availability of computer code

Data collection	gnomAD (v4.1)<br>ClinVar (accessed November 13th, 2022)<br>OMIM (accessed April 29th, 2024)<br>ACMG (v3.2)<br>Open Targets (v23.12)<br>Genebass (static)
Data analysis	Variant calling pipeline: https://broadinstitute.github.io/warp/docs/Pipelines/Exome_Germline_Single_Sample_Pipeline/<br>Joint genotyping pipeline: https://broadinstitute.github.io/warp/docs/Pipelines/JointGenotyping_Pipeline/<br>Ensembl Variant Effect Prediction (v105) with LOFTEE plugin (v1.04_GRCh38):<br>PLINK (v2.0) for basic operations on genotype data: https://www.cog-genomics.org/plink/2.0/<br>REGENIE (v3) for association testing: https://rgcgithub.github.io/regenie/<br>TREtools for trait extraction and preparation: https://github.com/genes-and-health/tre-tools<br>Custom codes for phasing and identifying biallelic genotypes: https://github.com/BRaVa-genetics/snakemake_pipeline_for_phasing<br>Custom codes for recessive association and other analyses: https://github.com/giorkala/gnh_flagship_recessive

For manuscripts utilizing custom algorithms or software that are central to the research but not yet described in published literature, software must be made available to editors and reviewers. We strongly encourage code deposition in a community repository (e.g. GitHub). See the Nature Portfolio guidelines for submitting code & software for further information.

Data

Policy information about availability of data

All manuscripts must include a data availability statement. This statement should provide the following information, where applicable:

- Accession codes, unique identifiers, or web links for publicly available datasets
- A description of any restrictions on data availability
- For clinical datasets or third party data, please ensure that the statement adheres to our policy

Summary-level data from the G&H 44,028 exomes are publicly available in a Google cloud storage bucket: https://console.cloud.google.com/storage/browser/genesandhealth_publicdatasets/results_44k_ExWAS for web access and gs://genesandhealth_publicdatasets/ for programmatic access. Individual-level data are only available within a Secure Data Environment with controlled access due to their sensitive nature. Bona fide researchers may obtain access upon application to G&H and approval by the Executive Committee. Detailed instructions can be found at https://www.genesandhealth.org/researchers/apply-for-access/.

Research involving human participants, their data, or biological material

Policy information about studies with human participants or human data. See also policy information about sex, gender (identity/presentation), and sexual orientation and race, ethnicity and racism.

Reporting on sex and gender	Provided in the manuscript.
Reporting on race, ethnicity, or other socially relevant groupings	Provided in the manuscript.
Population characteristics	Adult British volunteers of self-reported South Asian ancestry. The median age of recruitment was 39 and 56% were females among the 44,028 participants analyzed in the study. More details can be found in the cohort profile published previously (https://doi.org/10.1093/ije/dyz174).
Recruitment	British Bangladeshi and Pakistani individuals aged 16 and over are invited for voluntary participation. Recruitment largely took place in community settings or health care settings. More details can be found in a prior cohort profile paper (https://doi.org/10.1093/ije/dyz174). Self-selection, convenience sampling, or health status biases may limit the generalizability or the transferability of the findings.
Ethics oversight	The study was approved by the London South East NRES Committee of the UK Health Research Authority (14/LO/1240).

Note that full information on the approval of the study protocol must also be provided in the manuscript.

Field-specific reporting

Please select the one below that is the best fit for your research. If you are not sure, read the appropriate sections before making your selection.

☒ Life sciences ☐ Behavioural & social sciences ☐ Ecological, evolutionary & environmental sciences

For a reference copy of the document with all sections, see nature.com/documents/nr-reporting-summary-flat.pdf

Life sciences study design

All studies must disclose on these points even when the disclosure is negative.

Sample size	Up to 44,028 pending on the availability of the relevant phenotype data (not determined a priori).
Data exclusions	No exclusions except for predefined standard QC metrics as described in the Methods and the Supplementary Methods.
Replication	All significant associations found in this study were examined for replication in two independent datasets as described in the manuscript.
Randomization	Not relevant as this is an observational study.
Blinding	Not relevant as this is an observational study.

Reporting for specific materials, systems and methods

We require information from authors about some types of materials, experimental systems and methods used in many studies. Here, indicate whether each material, system or method listed is relevant to your study. If you are not sure if a list item applies to your research, read the appropriate section before selecting a response.

Materials & experimental systems

n/a	Involved in the study
☒	☐ Antibodies
☒	☐ Eukaryotic cell lines
☒	☐ Palaeontology and archaeology
☒	☐ Animals and other organisms
☒	☐ Clinical data
☒	☐ Dual use research of concern
☒	☐ Plants

Methods

n/a	Involved in the study
☒	☐ ChIP-seq
☒	☐ Flow cytometry
☒	☐ MRI-based neuroimaging

Plants

Seed stocks

Report on the source of all seed stocks or other plant material used. If applicable, state the seed stock centre and catalogue number. If plant specimens were collected from the field, describe the collection location, date and sampling procedures.

Novel plant genotypes

Describe the methods by which all novel plant genotypes were produced. This includes those generated by transgenic approaches, gene editing, chemical/radiation-based mutagenesis and hybridization. For transgenic lines, describe the transformation method, the number of independent lines analyzed and the generation upon which experiments were performed. For gene-edited lines, describe the editor used, the endogenous sequence targeted for editing, the targeting guide RNA sequence (if applicable) and how the editor was applied.

Authentication

Describe any authentication procedures for each seed stock used or novel genotype generated. Describe any experiments used to assess the effect of a mutation and, where applicable, how potential secondary effects (e.g. second site T-DNA insertions, mosiacism, off-target gene editing) were examined.
